# Aquaporins in the Spinal Cord

**DOI:** 10.3390/ijms17122050

**Published:** 2016-12-07

**Authors:** Michal K. Oklinski, Mariusz T. Skowronski, Agnieszka Skowronska, Michael Rützler, Kirsten Nørgaard, John D. Nieland, Tae-Hwan Kwon, Søren Nielsen

**Affiliations:** 1 Department of Health Science and Technology, Aalborg University, 9220 Aalborg, Denmark; mko@hst.aau.dk (M.K.O.); miru@hst.aau.dk (M.R.); kj12@student.aau.dk (K.N.); jdn@hst.aau.dk (J.D.N.); 2 Department of Animal Physiology, University of Warmia and Mazury in Olsztyn, 10-752 Olsztyn, Poland; skowron@uwm.edu.pl; 3 Department of Human Physiology, University of Warmia and Mazury in Olsztyn, 10-752 Olsztyn, Poland; agnieszka.skowronska@uwm.edu.pl; 4 Department of Biochemistry and Cell Biology, School of Medicine, Kyungpook National University, Taegu 41944, Korea

**Keywords:** aquaporin, spinal cord, astrocytes, neurons, endothelial cells, central nervous system, peripheral nerve

## Abstract

Aquaporins (AQPs) are water channel proteins robustly expressed in the central nervous system (CNS). A number of previous studies described the cellular expression sites and investigated their major roles and function in the brain and spinal cord. Among thirteen different mammalian AQPs, AQP1 and AQP4 have been mainly studied in the CNS and evidence has been presented that they play important roles in the pathogenesis of CNS injury, edema and multiple diseases such as multiple sclerosis, neuromyelitis optica spectrum disorders, amyotrophic lateral sclerosis, glioblastoma multiforme, Alzheimer’s disease and Parkinson’s disease. The objective of this review is to highlight the current knowledge about AQPs in the spinal cord and their proposed roles in pathophysiology and pathogenesis related to spinal cord lesions and injury.

## 1. Introduction

Since their discovery, aquaporin (AQP) water channels have gained a lot of attention. Their prominent expression and important function have been identified in several organs in the body, including kidney, salivary glands, muscle, lungs and central nervous system (CNS). AQPs display high capacity as well as selectivity for transporting water molecules across cell membranes [1,2,3,4]. AQPs are highly conserved and often classified into classical AQPs selectively permeable to water (AQP0, 1, 2, 4, and 5) and aquaglyceroporins with permeability to water, glycerol, and urea (AQP3, 7, 9, and 10) [5,6]. Furthermore, another group of so-called unorthodox AQPs has been distinguished, since their physiological roles are not well characterized (AQP6, 8, 11, and 12) [7].

The function of AQPs for osmotic water transport is influenced by the structural hourglass form. At the aromatic/arginine filter, the narrowest region of the pore (~2.8 Å), molecules larger than water are excluded from passage through classical AQPs [8,9]. AQP monomers are built up by six membrane spanning helical domains that surround this aqueous pore and are super-assembled in membranes as tetramers. Thereby, in contrast to many ion channels like potassium or cyclic nucleotide-gated channels where the pore resides at the center of the tetramer, each monomer acts as a functioning water channel [10]. Moreover, AQP selectivity is enhanced by steric and electrostatic factors, since the monomeric helices placed on the outside face of the tetramer are hydrophobic, whereas the hydrophilic ones are positioned close to the tetramer center [11,12]. Interestingly, in aquaglyceroporins the narrowing of the pore is less constricted (~3.4 Å) and lined by more hydrophobic residues [13].

In the CNS, mRNA amplification of AQP1, 3, 4, 5, 8, 9 and 11 was reported [14,15]. However, most studies in the brain or spinal cord have been limited to AQP1, 4 and 9 [16,17]. The most extensively studied site of AQP expression in the brain is the ventricular system (Table 1). Choroid plexus, enclosed in brain ventricles shows high abundance of AQP1 at the apical surface of the epithelial cells [16]. Therefore, this channel is involved in the secretion of cerebrospinal fluid (CSF) [18]. The cerebrovascular endothelium in the CNS lacks AQP1 [16,19] with the exception of the blood vessels in circumventricular organs [20] and small capillaries occasionally found in the brain and spinal cord parenchyma [20,21,22]. Outside of the brain, AQP1 expression was found in the dorsal horns of the spinal cord, in the dorsal root ganglia and its projecting nerves, particularly in the sciatic nerve (Table 1) [14,21,23]. A portion of AQP1 labeling in spinal cord was assigned to a population of the primary sensory neurons with small-diameter which are important in nociception and pain processing [21,23]. Moreover, other studies suggested a role of this water channel in the perception of inflammatory thermal pain and cold pain perception [24,25].

AQP4 is a water channel that is widely expressed throughout the CNS, particularly in astrocyte processes building up the glial-limiting membrane and astrocytes that are in contact with endothelial cells of the blood brain barrier (BBB) (Table 1). Furthermore, the presence of AQP4 was reported in subependymal astrocytes and in the basolateral membrane of ependymal cells forming an interface between CSF and CNS [17,26]. Strong AQP4 expression at the borders between brain parenchyma and major fluid compartments, e.g., between brain ventricles and glia limitans externa or at the BBB, suggests that AQP4 functions in facilitating water flow into and out of the brain and spinal cord. Moreover, an increased AQP4 expression has been demonstrated in glioblastoma multiforme, suggesting it is also involved in malignant brain tumors [27]. Although water transport can occur to a certain extent through simple diffusion across the plasma membrane [28], studies utilizing AQP4 knockouts demonstrated great importance of this water channel in the pathogenesis of brain water imbalance and CNS injuries. In disease models of obstructive hydrocephalus, focal cortical-freeze injury, brain tumor and intraparenchymal saline infusion, AQP4-deficient mice showed more severe hydrocephalus, markedly increased intracranial pressure, limited water elimination and poor neurological scoring when compared with their wild-type corresponding controls [29,30,31]. In contrast, several other reports concluded that AQP4-knockout mice possessed better survival rate and improved neurological outcomes compared with wild-type controls exposed to cytotoxic brain edema [32,33]. Complicated temporal and dimensional expression of AQP4 in different disease conditions with different onset time of injury make it difficult to distinguish between beneficial and disadvantageous roles of AQP4, since it may concurrently facilitate edema fluid clearance in one area of the brain while intensifying accumulation of water in another region [33,34,35].

In addition, it was also demonstrated that AQP9 is expressed in the murine CNS (Table 1), especially within midbrain catecholaminergic neurons [36] and rat subset of dopaminergic neurons and astrocytes [37]. However, another study exploiting *Aqp9* gene knockout mice found no evidence of AQP9 expression in the brain [38]. The objective of this article is to review the current knowledge regarding spinal cord AQPs expression in normal and pathological conditions, in order to analyze their possible function and pathophysiological importance as well as to explore novel research areas involving AQPs in the spinal cord and brain.

## 2. Expression Pattern of Aquaporins (AQPS) in the Healthy Spinal Cord

### 2.1. Aquaporin 1 (AQP1)

**Figure 1:**
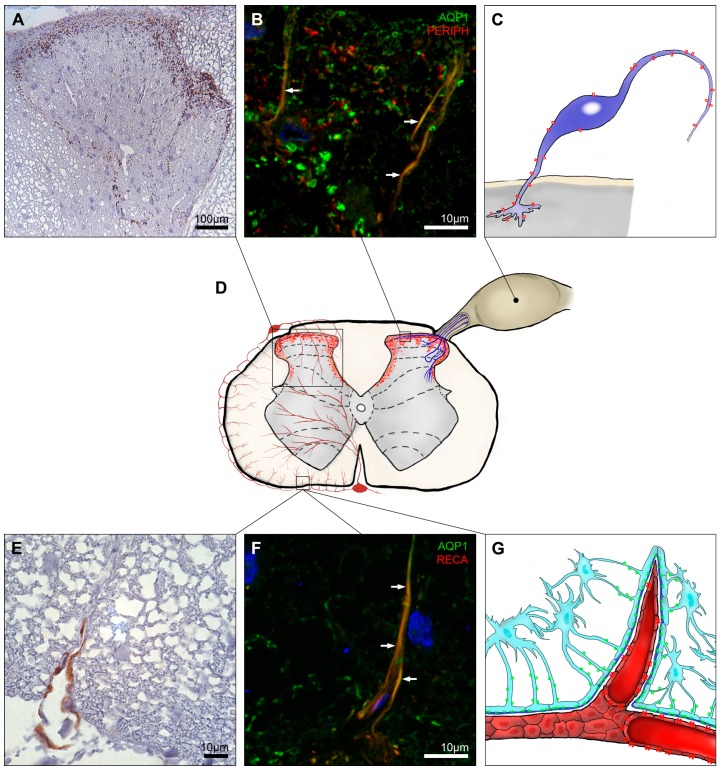
AQP1 expression in the spinal cord. AQP1 was strongly expressed at laminae I and II of the dorsal horn with decreasing signal intensity at the medial edges of dorsal horns up to lamina V (**A**,**D**); A fraction of the AQP1 signal belonged to unmyelinated neuronal cells (**B**) protruding from dorsal root ganglion to the superficial laminae of dorsal horns which axons build up peripheral sensory fibers (**C**); Illustration of AQP1 expression in the spinal cord cross-section (**D**); AQP1 labeling in the white matter was rather infrequent and found in proximity to the glia limitans (**E**) most likely belonging to small arterioles furcating from the arterial vasocorona (**F**,**G**). Panels **A**, **B**, **E** and **F** are modified from Oklinski et al. [21]. The blue signal in panels **B** and **F** represents 4′,6-diamidino-2-phenylindole (DAPI) staining of the nuclei; AQP1, aquaporin 1; PERIPH, peripherin and RECA, rat endothelial cell antigen-1. Colocalization indicated by arrows. AQP1 is indicated in red on panels **C**, **D** and **G**. AQP4 is drawn in green on panel **G**. White arrows in **B** and **F**.

AQP1 expression in the brain was mostly assigned to epithelial cells of choroid plexus [16,18], whereas the labeling pattern of AQP1 in the spinal cord is different. Essentially, AQP1 expression at cervical, thoracic and lumbar level of the spinal cord posesses a very uniform pattern. The dorsal horn in laminae I and II is especially rich with AQP1 spotted labeling (Figure 1A,D). AQP1 immunolabeling in the dorsal horns was mostly assigned to the unmyelinated sensory fibers with small diameter [24,39] (Figure 1B), and very few myelinated neurons [23]. Intense AQP1 labeling was found in synaptical terminal membranes, but not in the area of synaptic density [23] (Figure 1C). In laminae III and IV, AQP1 was dominantly present at the lateral and medial boundaries of the dorsal horns and in lamina V, a tangled, filamental pattern of AQP1 immunolabeling was seen [21]. In contrast, in other parts of gray matter AQP1 was pretty scarce with slightly increased expression in the immediate surroundings of the central canal [14,21].

Shields et al. [23] reported complete lack of AQP1 mRNA in the spinal cord and concluded that all AQP1 immunlabeling belongs to sensory neurons projecting form the dorsal root ganglion to the spinal cord dorsal horn (Figure 1C). AQP1 expression showed a 92% overlap with peripherin, a marker for small-diameter nociceptors in the dorsal root ganglion [25,56]. However, much less co-localization between these two proteins was observed in the spinal cord [21]. Therefore, further studies are necessary to precisely define the localization of AQP1 in the spinal cord dorsal horns.

Sparse and intermittent AQP1 labeling was found in immediate vicinity to the glia limitans at all levels of the spinal cord, which is apparently associated with small penetrating arterioles protruding from arterial vasocorona encountered in both white and gray matter (Figure 1E–G) [21]. These observations are in agreement with sporadical labeling of blood vessels in parenchyma of rat and human brains [20,22]. However, these observations seem to be exceptional since in general AQP1 is not expressed in the capillaries of neurovascular units and BBB [16,19,57]. Aside from these cases, one study reported AQP1 expression in astrocytes and ependymal cells in rat spinal cords [39].

### 2.2. Aquaporin 4 (AQP4)

**Figure 2:**
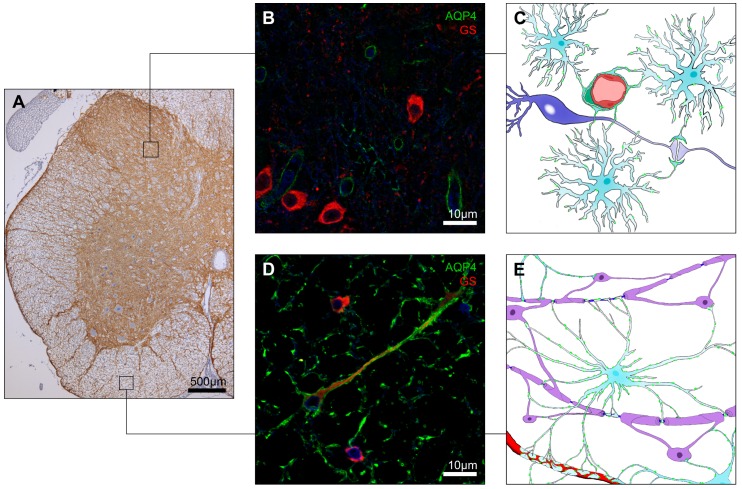
AQP4 expression in the spinal cord. AQP4 was abundantly expressed across the whole spinal cord with markedly higher intensity in the superficial lamina of dorsal horns, verges of ventral horns, glia limitans and surroundings of blood vessels (**A**); In the gray matter, protoplasmatic astrocytes AQP4 expression represents a polarized pattern with the highest intensity in the astrocytes foot processes encircling capillaries (**B**,**C**) and weaker labeling in processes belonging to the tripartite synapse (**C**); In the white matter, fibrous astrocytes AQP4 expression is observed along the cell membrane (**D**,**E**); Illustrations of AQP4 expression in the spinal cord (**C**,**E**) markings in green illustrate AQP4. The blue signal in panels **B** and **D** represents DAPI staining of the nuclei. Immunoperoxidase staining of AQP4 (**A**) and immunofluorescence images (**B**,**D**) are modified from Oklinski et al. [21]. AQP4, aquaporin 4; GS, glutamate synthase.

AQP4 labeling has been found in astrocytes of the white and gray matter along the whole spinal cord (Figure 2A). Fibrous astrocytes in spinal cord white matter are arranged in a radial pattern, with AQP4-labeled astrocyte processes stretching from gray matter to glia limitans (Figure 2A,D). Strong AQP4 immunolabeling is present in astrocytic end-feet encircling capillaries and building up the glia limitans (Figure 2A). In addition, substantial AQP4 signal was found in astrocyte processes enveloping myelinated neuronal fibers in longitudinal spinal cord white matter sections (Figure 2E) [43]. AQP4 signal within the white matter is also intense and colocalized with commonly used astrocyte markers, such as glial fibrillary acidic protein (GFAP) and glial glutamate transporter-1 (GLT-1) [14,21,44]. In gray matter AQP4 labeling was demonstrated in a distinctive laminar pattern with strong intensity in the laminae I and II of the dorsal horn (Figure 2A), whereas, in the deeper laminae, immunoreactivity decreased to moderate levels. However, labeling was slightly higher in lamina X surrounding the central canal, the medial section of the dorsal horn neck and at the thoracic level in the intermediolateral column. Moreover, in the gray matter more pronounced immunlabeling was detected around blood vessels (often in the form of complete rings encircling the blood vessels), neuronal perykarya and processes, especially in the superficial laminae of the dorsal horns (Figure 2B,C). A more filamentous and spotted pattern of AQP4 surrounding dendrites, neuropil and motor neurons was also observed in the ventral horns [14,21,44]. AQP4 labeling in spinal cord protoplasmatic astrocytes was slightly more polarized compared to white matter fibrous astrocytes (Figure 2C,E). Co-localization studies have shown less intense overlap between GFAP and AQP4, primarily in laminae I and II of dorsal horns and in perivascular end-feet [21,43]. On the other hand, GLT-1 colocalized with AQP4 especially in gray matter neuropil, at fine astrocyte protrusions around synaptic contacts, dendrites and perikarya [44].

There are two dominant AQP4 isoforms present in the CNS, M1 (323 amino acids) and the shorter M23 isoform (301 amino acids). Both types form homo- and hetero-tetramers and are highly water permeable [45,58]. The M23 isoform is required for the formation of higher order structures, known as square arrays [46,47]. Square arrays are predominantly found at astrocytic end-feet that form a continuous sheet surrounding blood vessels and glia limitans in the CNS [17,48]. In the protoplasmatic astrocytes of the spinal cord gray matter, AQP4 square arrays were found mostly in perivascular regions and their distribution resembles that reported in the brain cortical gray matter. However, in white matter, sizable clusters of AQP4 were observed in both parenchymal and perivascular regions. Moreover, both white matter regions possess similar numbers of AQP4 square arrays [43]*.*

A relevant aspect of AQP4 and other AQPs expression in different parts of CNS are potentially due to species differences in human and rodent astrocytes. Compared to mice, rat astrocytes express higher levels of intermediate filament proteins such as GFAP, nestin and vimentin. Furthermore, S100β a glial-specific calcium binding protein that is frequently used as an astrocyte marker, is expressed at higher levels in rat astrocytes [59,60]. Moreover, rat astrocyte ultrastructure resembles the structure of human astrocytes more closely than their murine equivalents [61,62], which needs to be considered when analyzing and comparing AQPs expression data between different species.

### 2.3. Aquaporin 9 (AQP9)

As in the brain, expression of AQP9 in spinal cord is still a matter of ongoing research. Earlier studies provided data about distinct AQP9 immunolabeling in ependymal cells, tanycytes and neurons in the rat brain [49,50], and astrocytic processes bordering the subarachnoid space and ventricles in the mouse brain [51]. In the spinal cord, immunolabeling was observed in particular in white matter and glia limitans astrocyte processes [14]. An ultrastructural analysis of rat brain performed by Amiry-Moghaddam et al. [37] suggested AQP9 expression in mitochondrial inner membrane of astrocytes and dopaminergic neurons in the substantia nigra and ventral tegmental area. However, as was found in the brain, immunolabeling in the spinal cord was undetectable when suitable positive and negative controls i.e., *Aqp9* gene knockout mice were used [38]. Subsequent detailed studies in rats and knockout mice conducted by Mylonakou et al. [52] reported limited presence of both AQP9 mRNA and protein in mouse and rat brains in neuronal cells, but expression in astrocytes remained elusive, because of nonspecific affinity of the utilized anti-AQP9 antibody to GFAP. The localization in neurons was confirmed in later reports, where the data of astrocytic AQP9 expression were acquired with the same antibody which Mylonakou had used [40]. Moreover, it was also demonstrated that AQP9 mRNA levels in the brain are substantially lower compared to the expression in liver. Furthermore, the observed expression level in mouse brain is about six times lower compared to rat brain [52]. Recent systemic data gathered from human samples revealed that AQP9 mRNA is expressed at very low levels in brain [53]. Despite some progress made for clarifying the true expression of AQP9 in the brain, the data on the potential presence and localization of this aquaglyceroporin in spinal cord remain limited. Ubiquitous expression of this channel has been suggested in fibrous white matter of spinal cord astrocyte processes, reaching to glia limitans, central canal and dorsal horn [14,40]. However, these reports need to be treated cautiously, considering that AQP9 expression in brain astrocytes also remains controversial.

## 3. AQP Expression in Disease Conditions of Spinal Cord

### 3.1. Aquaporin 1 (AQP1)

#### 3.1.1. AQP1 in Spinal Cord Injury (SCI) and Edema

The number of reports studying the expression of AQP1 in disease conditions of the spinal cord is still quite limited. Nonetheless, two studies described a significant up-regulation (up to eight-fold increase) of AQP1 in response to SCI at the injury site, with gradually decreasing expression extending into the caudal and rostral direction [39,63]. AQP1 up-regulation was detected as early as 6 h after injury [64] and persisted for up to 11 months [39]. AQP1 expression post injury was assigned to the membranes of dorsal horn neuronal fibers placed below laminae I and II, surviving spinal neuronal cell bodies and motoneuronal cells, reactive scar-forming astrocytes surrounding the lesion site and central canal ependymal cells [39].

SCI is commonly associated with blood-spinal cord barrier (BSCB) disruption, leading to vasogenic spinal cord edema manifesting in the accumulation of excess water in the extracellular space. However, spinal cord edema might be caused by a number of other factors. Li et al. [65] have used the ischemia-reperfusion injury model to study spinal cord edema. They reported that AQP1 expression is significantly up-regulated in astrocytes in the first 12 h after injury, which was correlated with early cytotoxic edema, whereas later up-regulation was observed in endothelial cells, which was associated with both cytotoxic and vasogenic edema [65].

#### 3.1.2. Changes of AQP1 Expression in Spinal Cord and Dorsal Root Ganglion in Response to Peripheral Nerve and Tissue Damage

Peripheral nerve injury (PNI) provides an interesting model to study the changes of AQP expression in the spinal cord. In a model of complete sciatic nerve transection, increased AQP1 expression was reported in the medial superficial part of the ipsilateral dorsal horn a week after surgery [23]. Similarly, in a sciatic nerve cut injury model highly elevated AQP1 immunolabeling was observed 24 h after the injury in the ipsilateral dorsal root ganglion, as well as in the dorsal and ventral horn of the spinal cord [66]. A similar increase in AQP1 signal intensity was reported following sciatic nerve crush injury [66]. In contrast, in a chronic constriction injury model of the sciatic nerve, Buffoli et al. [67] reported that the expression level of AQP1 in spinal cord and dorsal root ganglion was not altered. Moreover, no alterations in AQP1 level in spinal cord were found in a model of tissue damage where complete Freund’s adjuvant was intraplantary injected into a hindpaw [23].

#### 3.1.3. AQP1 in Multiple Sclerosis (MS) and Neuromyelitis Optica Spectrum Disorders (NMOsd)

NMOsd is characterized by longitudinally extensive transverse myelitis (LETM) with or without optic and spinal cord manifestation. It is associated with an appearance of IgG antibody (AQP4-IgG) targeted to AQP4-expressing astrocytes which distinguishes NMOsd from multiple sclerosis (MS) [68,69]. Additionally, several studies also suggested an emerging role of AQP1 in NMOsd. Autoantibodies against AQP1 (AQP1-Abs) were found among patients in the high risk group (characterized by presence of AQP4 autoantibodies with early forms of LETM or optic neuritis [70]) and diagnosed with NMO and/or MS [71,72,73]. Interestingly, patients with sero-positivity for AQP4-IgG belonging to the NMOsd group had higher reactivity to AQP1 peptides compared to the NMOsd group with AQP4-IgG sero-negativity [71,74]. AQP1-Abs found among NMOsd patients belong to the complement-activating IgG1 subclass that possesses affinity for the extracellular domain of AQP1 [72].

In a study by Tzartos et al. [72], five out of 42 MS patients produced AQP1-Abs and two out of these five had spinal cord lesions. In the same study, 17 of 22 AQP1-Abs sero-positive NMOsd patients were diagnosed with LETM and five of them were also co-diagnosed with NMO. Misu et al. [75] analyzed AQP1 and AQP4 immunoreactivity in the specimens of brains and spinal cords collected from 19 patients diagnosed with NMO or MS. They described six different lesion types present in the spinal cords from patients with NMO, where AQP1 and AQP4 were expressed in astrocytes in a distinct pattern. These lesions were localized in brain stem and spinal cord and were classified on the basis of astrocyte loss and structural changes, axonal injury and demyelination, polymorphonuclear leukocyte aggregations and intensity of complement deposition. For instance, in type 1 NMO lesions, GFAP-positive astrocytes expressed AQP1 but AQP4 was undetectable. Lesions of type 2 were characterized by fluid-filled cysts, perivascular fibrosis and presence of macrophages. In type 3 lesions, found in the spinal cord white matter tracts, reactive astrocytes were strongly labeled with AQP1, whereas AQP4 labeling was lost or variable. Type 4 lesions were characterized by selective loss of AQP4 with normal AQP1 and GFAP expression and no clear signs of structural damage. Finally, astrocytic clasmatodendrosis in NMO type 5 and 6 lesions was associated with variable expression of both AQP1 and AQP4 and signs indicative of cell apoptosis. On the other hand, specimens from MS patients displayed glial scar patterns characteristic of MS lesions often containing sizable protoplasmatic multi-nuclear astrocytes. These cells had apparently lost the contact between perivascular endfeet and glia limitans and showed strong GFAP and AQP1 labeling [75].

### 3.2. Aquaporin 4 (AQP4)

#### 3.2.1. AQP4 in Spinal Cord Injury (SCI) and Syringomyelia

Aside from brain injury models, SCI is probably one of the most extensively investigated areas examining AQP4 in the CNS. Although currently most studies on SCI are based on rodent models, there is one study by Nesic et al. [76] describing AQP4 expression in injured human spinal cords. Human spinal cords from three patients suffering from contusion at cervical segments C2, C5/6 and C8, respectively, with survival time of one year for the first two cases and two years for the third case, were analyzed. In all specimens astrocytes showed markedly increased expression of AQP4 in the uninjured white matter. In this area GFAP expression was also retained. However, in close proximity to the primary site of trauma-related cysts, expression of AQP4 was sparse or undetectable. Nevertheless GFAP expression was persistent in this area, indicating that AQP4 expression was selectively decreased in the surviving astrocytes [76].

Similarly, AQP4-negative astrocytes were reported in rat SCI, but their presence was noted at an earlier time point after injury. In contrast to human SCI nearly all investigated astrocytes in rat SCI models were strongly positive for AQP4 in later phases (from one up to five months post injury) [76]. The shift of AQP4 expression was also described in another rat SCI model where initial down-regulation was gradually (after two weeks) changed into persistent up-regulation [77]. It was proposed that this shift of AQP4 expression may be related to astrocyte migration and that a significant increase in AQP4 expression could reflect astrocytes reaching the wound area. Furthermore, chronically injured spinal cords also showed increased AQP4 levels not only at the site of injury but along the whole spinal cord. This increase in AQP4 was observed throughout the nine-month study period [78]. Similarly, a significant increase in AQP4 immunoreactivity at 5 h, 24 h, three days and 14 days post SCI was also observed in a rat spinal cord compression model [79]. However, despite overall AQP4 up-regulation in chronically affected spinal cords, astrocytes forming the glia limitans externa exhibited markedly decreased AQP4 levels [77].

SCI models were frequently exploited to test potential therapeutic approaches by targeting changes of AQP4 expression. In most of these studies conducted in both rats [80,81,82,83] and mice [84,85], SCI contusion and compression [64,86,87,88,89] induced a common pattern of AQP4 up-regulation. These preclinical trials have recently been reviewed in detail by Yonan and Binder [90].

Posttraumatic syringomyelia is frequently a consequence of major and minor spinal cord injury manifested by the accumulation of CSF or fluid of different origin in cysts within the spinal cord (known as syrinxes) [91,92]. A study by Hemley et al. [93] has shown a relation between the increase of astrocytic AQP4 expression and the persistence of syrinxes in a rat model of posttraumatic syringomyelia where a significant up-regulation of AQP4 was observed at three and six weeks post injury at the level of the syrinx and in adjacent rostral and caudal levels.

In contrast to the frequently studied SCI models, there is only one report so far that describes AQP4 expression in the spinal cord after PNI [94]. Interestingly, sciatic nerve injury evoked a long lasting, two to threefold increase in AQP4 expression in the lumbar spinal cord that was associated with persistent enlargement of astrocyte processes. AQP4 up-regulation, an increase in astrocyte process volume, as well as length and number of branch points were correlated with development of pain [94].

#### 3.2.2. AQP4 in Neuromyelitis Optica (NMO) and Multiple Sclerosis (MS)

NMO is an autoimmune disorder characterized by the presence of AQP4-targeted autoantibodies. This novel class of autoimmune channelopathy [95] was subsequently distinguished from NMOsd [70]. A distinctive feature of NMO is a deficiency in AQP4 expression, especially surrounding vessels in the brainstem and spinal cord. The affected areas show prominent eosinophil infiltration and vascular accumulation of IgM and IgG [96,97,98]. Furthermore, significant loss of AQP4 and GFAP has been described in astrocytes of cultured mouse spinal cord slices following exposure to NMO-IgG and complement. This treatment is known to result in NMO-like lesions with concurrent neuronal demyelination [99].

Another detailed description of AQP4 expression at NMO spinal cord lesions was provided by Misu et al. [75]. Lesions were characterized by variable loss of axons, oligodendrocytes, myelin, complement activation and inflammatory macrophage and T cell accumulation. Astrocytes in the area either contributed to fibrillary gliosis or presented with nuclear DNA damage or bundled and retracted processes. Thereby, AQP4 immunoreactivity varied from decreased to undetectable [75].

Presence of anti-AQP4 autoantibodies in the serum of patients diagnosed with NMO was positively correlated with the length of spinal cord lesions on magnetic resonance imaging (MRI) [100]. Although this correlation has not been found in a later study by Waters et al. [101], the presence of anti-AQP4 antibodies was confirmed in 76% of NMO patients. Furthermore, Zeka et al. [102] demonstrated in Lewis rats that T cells targeted to the AQP4_268–285_ epitope, together with NMO-IgG can cause severe lesions with damaged astrocytes localized mostly in the gray matter of the spinal cord at the cervical and thoracic level. Noteworthy, when large numbers of AQP4_268–285_ specific T cells (~2 × 10^7^) were applied to initiate lesions, this resulted in a loss of astrocytes and AQP4 and GFAP labeling throughout the brain and spinal cord. In contrast, when small numbers of AQP4_268–285_ specific T cells were used, this exclusively induced characteristic NMO-like lesions in the spinal cord [102]. Importantly, initiation of antibody-independent characteristic NMO-like lesions caused by AQP4-reactive T cells was observed in a mouse model [103]. Administration of AQP4-reactive T cells to healthy wildtype mice induced infiltration of inflammatory CD3+ lymphocytes and demyelination predominantly in the spinal cord and brain. The described AQP4-reactive T cells were acquired by immunization of AQP4 knockout mice with human AQP4_56–69_, AQP4_135–153_, and AQP4_212–230_ extracellular loop peptides in complete Freund’s adjuvant followed by peptide targeted in vitro polarization of T cells into T_H_ 17 phenotype [103].

Although NMO has initially been considered as a subtype of MS, further studies revealed differences between the two diseases. The progression of MS and NMO lesions shows different characteristics with regard to astrocytes. Reduced AQP4 expression was mostly observed in cases of acute or chronic MS [104,105]. This reduction was linked to perivascular glia limitans disruption, which is characteristic for pattern III demyelination [104]. Enlarged protoplasmatic astrocytes with withdrawn or lost perivascular foot processes retained decreased or negative AQP4 immunoreactivity [75,104]. However, reactive astrocytes-forming MS glial scars close to the active MS lesions were associated with highly increased expression of AQP4 in the cell body and processes [75,97,106]. In the autoimmune encephalomyelitis-induced MS mouse model, the white matter in the spinal cord was characterized by axonal degeneration and demyelination, severe restructuring of neurovascular units and increased AQP4 immunoreactivity in the impaired fibrous astrocytes. In contrast, gray matter astrocytes appeared to be much less affected [107]. These data and detailed NMO findings described above suggest that changes of AQP4 expression in astrocytes in the lesions of MS are co-occurring events and not the primary disease target, even though both are immune-mediated conditions, resulting in demyelination and axonal loss [97].

#### 3.2.3. AQP4 in Amyotrophic Lateral Sclerosis (ALS)

ALS is another pathology where AQP4 expression changes and lesions of degenerated motor neurons in motor cortex, corticospinal tracts, brain stem and spinal cord leading to muscle paralysis [108]. Well documented features of ALS lesions are swollen and degenerated astrocytic foot-processes surrounding neurovascular units and lining the glia limitans with markedly increased expression of AQP4 and GFAP [109,110]. In the superoxide dismutase 1 (SOD1) mutant rat model of ALS, a detailed analysis of spinal cord tissue sections revealed AQP4 up-regulation in the immediate vicinity of motoneuron perikarions, which was absent in control animals [109]. Furthermore, decreased expression of Kir4.1 channels in affected ALS astrocytes [111,112], was associated with their decreased ability to buffer excess of K^+^ ions [111]. Impaired capacity of astrocytes to eliminate excessive K^+^ ions together with defective glutamate clearance [113,114] is likely contributing to the degeneration and death of motor neurons in ALS.

An additional finding is the correlation between disruption of the BBB and an increase in AQP4 in the perivascular astrocytes in the ALS models, where AQP4 was postulated as a marker for BBB integrity [115,116,117]. Moreover, studies in animals have shown that signs of BSCB disruption, like diminished levels of tight junction proteins, perivascular edema with swollen astrocytes foot processes and leakage of Evans blue from spinal cord capillaries can precede ALS-related neuroinflammation [118,119,120]. Nevertheless, the exact causes of ALS-related BBB and BSCB damage are still unknown.

## 4. Implications of Physiological and Pathophysiological AQP Expression in the Spinal Cord

AQP expression and function in physiology and pathophysiology of the brain has been thoroughly studied, whereas the role of these water channels in the spinal cord remains less well characterized. It is noteworthy that the expression of AQPs in the spinal cord is not identical to the expression in the brain (Table 1). Furthermore, recent advancement points to the critical roles of water channels in the pathogenesis of spinal cord injuries, including edema formation and development of persistent pain. Thus, further studies are warranted to examine the critical role of AQPs in disease conditions of the spinal cord.

Altered expression of AQPs in the spinal cord shows similarity in disease conditions. The changes include the loss of polarity in protoplasmic astrocytes, which is often associated with withdrawal of foot processes ensheathing the blood vessels. Other astrocytes become more reactive with increased AQP4 expression and swelling of processes. Interestingly, in most cases, AQP4 expression is up-regulated with varying temporal characteristics, whereas only in NMO AQP4 expression is diminished, which appears to be the direct disease target.

Changes in AQP expression in spinal cords can affect glutamate uptake and K^+^ buffering in astrocytes and threaten the electro-chemical homeostasis of active neurons [121,122]. In normal physiology, astrocytes can protect neurons from an excess of glutamate by converting it into glutamine and thereby avoiding glutamate-induced cytotoxicity [123,124]. A number of associations that could affect neurons by altering the water and ionic imbalance have been reported. For example, Cl^−^ and K^+^ channels, such as two-pore domain potassium channels, volume regulated anion channels or Na^+^-K^+^-Cl^−^ cotransporters are known to be involved in astrocyte volume regulation [125]. In addition, down-regulation of glutamate transporter GLT-1 was described in AQP4 null mice [126] and glutamate-triggered increase in the water permeability of AQP4 was found in cultured astrocytes [127]. Notably, it is known that the local concentration of glutamate in the spinal cord can increase in response to PNI or SCI [128,129]. This may result in the up-regulation of both AQP1 and AQP4. Therefore, the cytotoxicity and necrosis reported in brain and spinal cords of SCI, NMO, ALS and MS models may at least in part be mediated by altered AQP expression, in addition to the different causes.

Loss of astrocyte polarity, in particular the loss of AQP4 in vasculature ensheathing astrocyte processes is another feature observed in animal models of Alzheimer’s disease [130], MS [131] and NMO [132], which is considered to be a sign of impaired BBB integrity. Likewise, damaged integrity of both, BBB and BSCB has also been reported in patients and animal models of ALS [117] and SCI [77], often in connection with down-regulation of tight junction proteins, degeneration of endothelial cells and withdrawal of swollen astrocyte foot-processes and spinal cord edema. Impairment of BBB and BSCB can facilitate penetration of inflammatory cytokines further exacerbating dysfunction and resulting in neuronal death. Therefore, a common role of AQP4 in determining astrocyte polarity and BBB as well as BSCB integrity requires further investigations.

Furthermore, another interesting element in spinal cord diseases is the up-regulation of AQP1 expression in astrocytes. In general, AQP1 expression in astrocytes has not been reported in healthy spinal cords and brains of rodents and humans [41]. Thus further studies are warranted to evaluate the significance of AQP1 up-regulation in astrocytes described in rodent models of SCI [39] and spinal cord edema [65], as well as in human patients diagnosed with NMO [75].

Overall, more studies are needed to define the regulatory mechanisms for AQP1 and AQP4 expression in normal and disease conditions in the CNS. Moreover, the expression of AQP1 in sensory fibers may suggest involvement in pain transmission. Additionally, the expression and potential function of other AQPs in both spinal cord and brain remains to be clarified.

## Figures and Tables

**Table 1:** Comparison of AQP1, AQP4, AQP9 and AQP11 expression between spinal cord and brain cells and structures.

	Spinal Cord		Brain	
Aquaporin (AQP)	Cell Type and/or Structure	Detection Method and Reference	Cell Type and/or Structure	Detection Method and Reference
AQP1	Unmyelinated sensory fibers in DH Myelinated neuronal fibers (sparse) in DH (#) Lamina V and X Endothelial cells of small penetrating arterioles in immediate vicinity to the glia limitans (sparse, #) Astrocytes and ependymal cells (#)	IHC, RT-PCR, WB [23,24,39] IHC [23] IHC [14,21] IHC [21] IHC [39]	Epithelial cells of choroid plexus Endothelial cells of circumventricular organs Endothelial cells in blood vessels in brain parenchyma (sparse, #) Astrocytes, schwann cells surrounding the oculomotor and trigeminal cranial nerve fibers, neurons on the surface of the pia blood vessels (#)	WB, IHC, IMEM [16,18] IHC, RT-PCR [20] IHC [20,22] IHC [40,41,42]
AQP4	Astrocytes end-feet encircling capillaries, and building up the glia limitans, processes enveloping myelinated neuronal fibers; Expression polarized to foot-processes in protoplasmatic astrocytes and more evenly distributed in fibrous astrocytes	IHC, RT-PCR, WB [14,21,43,44]	Subpial astrocytes processes forming glia limitans, perivascular astrocyte endfeet in cortex; expression highly polarized to astrocytes foot-processes Basolateral membrane of ependymal cells	IHC, IMEM, NB, RT-PCR, WB, [17,26,43,45,46,47,48] WB, IHC, IMEM [17,26]
AQP9	Astrocyte processes in white matter and glia limitans (#)	IHC, RT-PCR, WB [14,40]	Neurons, ependymal cells (#), tanycytes (#) Astrocytes mitochondria and astrocytes in the immediate vicinity to subarachnoid space and ventricles (#)	IHC, RT-PCR, WB [37,38,40,49,50,51,52,53] IHC, RT-PCR [36,37,38,52]
AQP11	Not investigated	Not investigated	Dendrites of Purkinje cells in cerebellum, neurons in hippocampus and cerebral cortex (#) Epithelium of the choroid plexus and at the endothelium of the brain capillary (#)	IHC, NB, RT-PCR, WB [15,54] IHC, RT-PCR, WB [55]

WB: Western blotting, IHC: Immunohistochemistry, IMEM: Immunoelectron Microscopy, NB: Northern blotting, RT-PCR: Reverse transcriptase polymerase chain reaction, DH: Dorsal horn. Data marked with **#** are not fully confirmed and require further investigation.
